# Redressing the Corporate Cultivation of Consumption: Releasing the Weapons of the Structurally Weak

**DOI:** 10.34172/ijhpm.2020.205

**Published:** 2020-10-27

**Authors:** Sharon Friel

**Affiliations:** School of Regulation and Global Governance, Australian National University, Canberra, ACT, Australia.

**Keywords:** Food Systems, Corporations, Power, Structure, Agency, Governance

## Abstract

Corporate control of the global food system has resulted in greater global availability of highly processed, packaged and very palatable unhealthy food and beverages. Environmental harm, including climate change and biodiversity loss, occurs along the supply chains associated with trans-national corporations’ (TNCs’) practices and products. In essence, the corporatization of the global food system has created the conditions that cultivate excess consumption, manufacture disease epidemics and harm the environment. TNCs have used their structural power – their positions in material structures and organizational networks – to establish rules, processes and norms that reinforce and extend the paradigm of the neoliberal corporate food system. As a result, policy and regulatory environments, and societal norms are favourable to TNC’s interests, to the detriment of nutrition, health and environmental outcomes. There is hope, however. Power, of which there is many forms, is held not just by the TNCs but by all actors concerned about and connected to the food system. This paper aims to understand these power dynamics, and identify how structurally weak, public-interest actors can release their agency and work to achieve positive structural change. Such an analysis will help understand how the status quo can be disrupted and healthy and sustainable food systems created. The paper draws from the health governance and social movement literature, examining the Doha Declaration on the Trade-Related Aspects of Intellectual Property Rights (TRIPS) Agreement and Public Health, the Framework Convention on Tobacco Control (FCTC), and the Divestment movement. These cases demonstrate the many ‘weapons of the weak’ that can, against all odds recalibrate structural inequities. There is no one approach to transforming the corporate food system to become a healthy and sustainable food system. It involves coalition building; articulation of an ambitious shared vision; strategic use of multi-level institutional processes; social mobilization among like-minded and unusual bedfellows, and organized campaigns; political and policy entrepreneurs, and compelling issue framing.

## Background

 What, when, where and how much food people consume is no accident. Consumption is structurally determined, evolving from political, economic, social and cultural forces within and beyond the food system. It would be incorrect however to believe that people, including nutrition, health and environmental interest groups, have no say in the functioning of these systems. Everyone has agency, including civil, market and state actors. A key question, which is the focus of this paper, is how to use that agency in the public interest, and create the necessary structural changes in the global food system to improve population nutrition and environmental outcomes.

Central to this question are issues of power and governance. Historically, governments were the lead actors who set the policy framework to which the spectrum of public and private actors adhere. However, the relationship between the state and society has changed significantly since the latter half of the twentieth century. Today, the processes of governing – defined here broadly as ‘authoritative social steering toward a collective goal’ – involve a diversity of actors and organizational forms, and span multiple sectors and levels.^1^ Governments share the activity of governing with industry, international organisations, non-governmental organisations (NGOs), and civil society groups. Each actor uses a range of political, institutional, and discursive strategies to articulate their interests, exercise their rights, influence norms, take decisions, and meet their obligations.

The starting assertion of the paper is that economic globalisation, marketization^2-5^ and the increased power and influence of trans-national corporations (TNCs) in food systems governance has profoundly altered the purpose and functioning of the global food system. The changes in control of different parts of the supply chain have seen increased penetration and concentration of transnational food and beverage manufacturers, retailers and fast food chains in national food environments.^6,7^ One consequence of these changes is much greater global availability of highly processed, packaged and very palatable unhealthy food and beverages.^8,9^ Another consequence is the environmental impact, including climate change and biodiversity loss that occur along the food supply chains associated with these TNCs’ practices and products.^10,11^ In essence, the corporatization of the global food system has manufactured the conditions that cultivate mal-consumption, resulting in poor nutrition, non-communicable diseases (NCDs), and environmental harms.

There is hope, however. Remembering that the governance of food systems involves a diversity of actors spanning multiple sectors and levels^1^ offers possibilities for change. Power, of which there is many forms, is held not just by the food TNCs but by all actors concerned about and connected to the food system. The focus of this paper is to understand these power dynamics, and identify how structurally weak, socially-oriented actors can work to achieve positive change. The research question is ‘how can different dimensions of power enable or constrain the transformation of food systems to be healthier and more sustainable?’

Harris et al note “a useful analytic strategy is to view power through relations between people as individual actors and collectives, and the systems and structures they live or work in.”^12^ The paper uses this analytical approach to explore agentic power held and used by market, state, and civil actors; structural power embedded in institutional norms, rules and processes, and the power of ideas and discourse.

Agentic approaches to power emphasize the influence of an actor over or with other actors.^13^ Actors can work alone or as collectives who share resources and similar goals.^14^ Structures are the “rules, mandates and norms that influence lines of command, divisions of labour, resources, responsibility and channels of communication.’^15^ Structural power allocates differential capabilities to actors, enabling them to control outcomes based on their ability to shape the rules of the system. It also shapes actors interests and their ideology. Ideas mediate between structures and actors. Ideational power is ‘the capacity of actors to influence actors’ normative and cognitive beliefs through the use of ideational elements (eg, symbols, narratives, frames).’^16^ These elements form discourses, which are communicated in institutions and by actors to shape the problem and solutions, and become embedded in institutional structures and practices. Exploring discursive, institutional and structural dimensions of power helps understand actor-to-actor relations, the ways in which ideas and discourse are used as important forms of influence,^16^ and how actors use and shape institutional norms, rules and processes in ways that enable particular courses of action.^14,15,17^

 After briefly canvasing the literature associated with the rise of the corporate food regime, the paper applies the analytical power framework to examples from the health governance and social movement literature to demonstrate the different forms of power and strategies used by public interest actors to counter the interests and influence of TNCs. Three cases are examined, selected to illustrate the dynamics of power among different mixes of state and non-state actors; in health and non-health policy domains; and through the use of hard and soft regulatory approaches. The focus of each case is at the global level, and does not examine national or sub-national power dynamics and strategies.

 The first case is the Doha Declaration on the Trade-Related Aspects of Intellectual Property Rights (TRIPS) Agreement and Public Health [the Declaration]. This is an example of multisectoral state and non-state actors organising around a campaign focused on access to medicines, with the aim of reforming an economic (ie, non-health) policy instrument (trade agreement). The result reduces the power of Big Pharma. The second case is the Framework Convention on Tobacco Control (FCTC). Here, state and non-state actors organise around tobacco control by focusing on the development of an international health policy instrument (the treaty) to regulate Big Tobacco. The third case is the Divestment movement. This case focuses on non-state actors and their campaign for divestment from fossil fuel industries. While there is no focus on a specific policy instrument, the ultimate aim is to regulate the fossil fuel industry and change government policy.

 The final section of the paper discusses lessons from these cases, offering hope that collective agency can bring social interests, including nutrition and environmental goals, into decision-making processes throughout the food system. The section situates these lessons within a governance for healthy and sustainable food systems framework.

## Key Messages

**Implications for policy makers**

- Political commitment must be courageous in its support to address the powerful interests associated with the commercial drivers of unhealthy and unstainable food systems.
- Policy-makers have agency to improve food systems through the use of regulatory instruments.
- Strategic use of policy-makers institutional power is essential, and involves intergovernment mechanisms, stakeholder consultations and collaboration with non-governmental organisations (NGOs).

**Implications for public**

Redressing the corporate cultivation of consumption will protect the public from premature death and diet-related illnesses and protect the environment. Political courage is needed to implement the regulatory policies that are needed. Such courage will likely be more forthcoming where a vibrant civil society demands action. Structural reform of the global food system requires the strategic use of institutional and discursive power, requiring coalition building including among concerned citizens; articulation of an ambitious shared vision; social mobilization among like-minded and unusual bedfellows, and organized campaigns; political and policy entrepreneurs, and compelling issue framing.

## The 21st Century Corporate Global Food Regime

By the start of the 21st century, a handful of TNCs dominated the global food system. Ten food processors and manufacturers controlled 37.5% of the global market share of the world’s top 100 food companies; Nestle, Pepsi-Co and Kraft are the top three most proﬁtable ﬁrms that manufacture agricultural products into food products.^18^ In 2015, Coca Cola had just under 50% of global carbonated soft drink sales.^19^

Concentrated corporate power influences people’s nutrition and the environment by creating greater availability, affordability, and palatability of resource-intense, highly processed and packaged foods.^20-23^ Consolidation of supply chains means that TNCs can increase their market penetration, flood markets with the foods that they produce and manufacture, and in doing so, displace any healthier local alternatives. Concentrated power also increases food price due to lack of competition – small retail outlets are no longer able to compete in terms of retail prices or afford commercial rents as a result of TNCs which locate close by and drive up costs. Because of their size and generation of very significant profits, TNCs can devote large amounts of money to marketing, eg, Coca-Cola spends approximately $4 billion on marketing each year, which is more than the public health budget for many low-income and middle-income countries. This increases the global reach of the TNCs, and their ability to convince millions, if not billions, of people to want to purchase and consume their products.^24^

###  The Many Faces of Trans-national Corporation Power

 How did this happen? How have TNCs risen to shape the global food system and alter consumption practices so profoundly? How have they become legitimate actors in the national and global governance of food systems? The supply chain consolidation and market concentration of these TNCs was both a cause and consequence of their structural power, amplified by the use of institutional and discursive strategies.

Throughout the course of the late 20th century, corporations used their positions in material structures and organizational networks^25^ to establish new rules, processes and norms that reinforced and extended the paradigm of the neoliberal corporate food system. In the 1980s the control of access to capital moved beyond national governments to global financial institutions including the World Bank and the International Monetary Fund, and global corporations.^26^ These institutions created the political and economic structures that made countries amenable and pliable to the penetration of TNCs.

The global trade and investment system was one very important mechanism of this and is a policy arena in which TNCs promote free market ideas concerning greater liberalisation and deregulation.^27^ Institutionally, industry actors have much more access to the trade negotiators. This occurs through formal and informal mechanisms such as staying in the same hotel during negotiation rounds, inviting negotiators to trade industry dinners, and the revolving door phenomenon where industry executives move between corporate posts and trade negotiator positions.^28^ The result is trade agreements that favour private sector interests, which has major implications for nutrition and the environmental risks.^29-33^

TNCs also exercise their structural power via networks, specifically food industry alliances. For example, many food TNCS are members of the International Food and Beverage Alliance, which is an international lobby group made up of eleven of the world’s largest food companies. The International Food and Beverage Alliance endeavours to control the global nutrition debate, keeping it focused on under-nutrition in developing countries, with fortiﬁed food products providing solutions.^23^

Public private partnerships are important institutional mechanisms that TNCs use to shape the policy agenda and regulations, at national and international levels. Through engaging in public-private-partnerships, TNCs have access to policy decision-making processes, enabling them to influence the choice of issue or area for which rules and regulations are designed, or not, as well as the actual design, implementation, and enforcement of these rules.^34^

Discursive power has been important in creating the food regime shift towards a corporate-controlled food system. This involved controlling the narrative, by planting ideas, using symbolism and metaphors, influencing perceptions and ultimately shaping norms and values.^35^ At the global level, financial institutions used the discourse of ‘poverty reduction’ and ‘improved global food security,’ when promoting market liberal policies including trade liberalization.^36-38^ TNCs have also been effective in their use of discursive power. Analysis of food industry submissions to the Trans Pacific Partnership Agreement negotiations found that the industry framed their comments in ways to suggest that only greater trade and investment liberalization could raise living standards and benefit the economy and country.^39^ In using this framing, TNCs promote neo-liberal ideas as the dominate policy approach, ensuring that alternatives were not considered.^40^

Through the use of metaphors, such as the “nanny state” in media advertising, op-eds, think tank talking points, and public relations campaigns, TNCs promote a political and public discourse highlighting freedom of choice and individual responsibility, thus working to undermine government regulation and promote private self-regulation – which in turn strengthens their structural power.^28^ Similarly, Clapp and Scrinis have shown how food manufacturers use the idea of ‘nutritionism’ – the reduction of food’s nutritional value to its individual nutrients. By focusing on speciﬁc nutritional issues, these companies ‘establish themselves as leaders providing solutions to nutritional issues, and shift attention away from the broader health implications of the highly processed, and highly profitable, foods that they continue to manufacture and market.’^23^

## Releasing the Agency of the Structurally Weak: Learning From Multisectoral Policy Arenas

 Given the challenges enumerated in the previous section, can we identify any promising strategies that will curb the excesses of the current TNC-driven food system? Is there a way of effectively regulating the actions of the TNCs, and repurposing the global food system towards good nutrition and environmental outcomes?

Recalibrating the significant structural power differentials is vital for reconciling these tensions between corporatisation, consumption, nutrition and environmental sustainability, such that the interests of ‘weaker’ governments and social actors can be pursued. This requires fostering a process of collective actor agency and thorough structural reform, whereby people, organisations, and nations gain control over the decisions that affect them and their shared vision. Described below are some lessons from policy areas outside of nutrition that offer some insights into social mobilization for outcomes that defy apparently long odds. Each case illustrates forms of power operationalised through organised strategies, which harnessed political consciousness and pushed a collective vision.^41^

###  The Trade System, Pharmaceutical Companies, and Access to Medicines

The 1995 World Trade Organization’s (WTO’s) Agreement on TRIPS established new intellectual property protections for pharmaceutical products. Countries in the grip of an HIV/AIDS pandemic realized that these protections made the cost of patented medicines unaffordable. In 2001 WTO members adopted the Doha Declaration on the TRIPS Agreement and Public Health [the Declaration] that clarified that nothing in TRIPS should “prevent Members from taking measures to protect public health,” including the domestic manufacture of cheaper generic medicines.^42^

Widely seen as a success for access to medicines, the Declaration demonstrated the power of developing countries, supported by organised civil society, to drive through a health equity agenda, which was very different to the interests of the pharmaceutical companies and large developed economies.^43^ There were various discursive and institutional strategies used, over a number of years, by developing countries and health advocates that were critical to the development of the Declaration.^27^

A coalition of health workers, HIV/AIDS activists and organizations launched a campaign for access to medicines in 1996. The campaign used different forms of discursive power, including the frame “copy = life” to advocate generic and affordable production of life-saving patented drugs.^44^ In 1998, thirty nine pharmaceutical companies, backed by the US, which was a powerful actor in the TRIPS negotiations, sued Mandela’s government for trying to make drugs affordable in South Africa.^45^ In response to this, health advocates used discursive frames such as ‘Gores greed kills’ to highlight the prioritization of profits over millions of dying patients.

The campaigners used institutional strategies including forum shifting. They first raised generic manufacture and trade tensions within the World Health Organization (WHO) as this was a more hospitable forum for such issues than the WTO. Through the WHO, the health campaigners worked with HIV/AIDS affected governments to develop what would become the Doha Declaration.^44^ In 1999, WHO’s governing body of member states, the World Health Assembly, unanimously approved what would become the Doha Declaration.

 Another important institutional factor in the access campaigner’s success was their collaboration with generic drug manufacturers in India. Enlisting market actors can be an important strategy to build support for a campaign, with advocates being able to capitalize on divisions within industries to shore up support.

The agenda then moved out of WHO into WTO. At the Doha round of WTO negotiations in November 2001, developing countries refused to negotiate unless the Doha Declaration was adopted first. Governments from developing countries demonstrated that by establishing a core coalition, and maintaining it throughout the negotiating process, they could prevent themselves from being out-maneuvered by the European Union-US block. The mobilization of a wider circle of countries within the negotiations that were supportive of the core countries was another important institutional strategy that contributed to the success. Also important was the visible support of the goal of the core group of developing countries by access campaigners plus other national and international civil society groups.^43^

###  Framework Convention on Tobacco Control and the Tobacco Industry

The establishment of the FCTC is an example of a health victory against TNCs, in this instance Big Tobacco. Since the 1950s, the tobacco industry, like the food industry, had intensified its structural and economic power, enabled by modern processes of globalisation.^46^ WHO and the global health community were very concerned, and recognised the need for a transnational approach to regulation to address the transnational nature of the tobacco epidemic. The resulting FCTC marked an important structural change in global health governance. It was the first time that WHO had used its treaty-making powers (168 of the World Health Assembly’s 192 member countries signed the FCTC in 2003, becoming international law in 2005).^47^ By extending the debate about tobacco control beyond public health medicine to include trade, economics, and law, the FCTC reframed the solutions to tobacco control, and established rules of engagement with the tobacco industry [through Article 5.3].

The successful development of the FCTC required support from across multiple policy domains, and from state and non-state actors. The use of institutional and discursive power by governments and civil society groups was key to the successful development of the FCTC. Collins et al describe the use of inter-governmental processes within the WHO, with coalitions of WHO member states - the core constituency of the FCTC – quickly forming.^48^ At the 1999 World Health Assembly 50 member states took the floor to commit political and economic support for the proposed process to achieve the FCTC. Powerful regional support emerged eg, 21 countries of the African Region adopted the Johannesburg Declaration on the FCTC in 2001.

Cross-UN support was generated through improved inter-agency mechanisms, and the World Bank support for tobacco control provided credibility to the case for the FCTC. Notably, the Bank’s 1999 publication ‘Curbing the Epidemic,’ which concluded that tobacco control is good for health and the economy, influenced many countries during the FCTC negotiation, especially developing countries, where the World Bank wields enormous influence.^49^

 Civil society actors exerted their institutional power through formal and informal processes. International NGOs with ‘Official Relations’ status could formally participate in WHO proceedings. They attended all the Working Group and Intergovernmental Negotiating Body meetings, some as members of government delegations. In an attempt to enable input to the negotiations from a wider range of non-state actors, WHO held public hearings, receiving over 500 written submissions and testimonies from 144 organisations.

 NGO’s also created their own informal spaces to educate, mobilize, and engage state and non-state support for the FCTC. They prepared briefings for government delegates on technicalities of the proposed treaty; held policy discussions with governments; wrote letters to delegates; held press conferences before, during and after the negotiation meetings, ran an effective media campaign, and published reports about tobacco industry practices. The establishment of the Framework Convention Alliance (FCA) in 2000, a heterogeneous alliance of more than 60 civil society organisations and coalitions from around the world, enabled better communication and resource sharing among NGOs and health activists in developed and developing countries. An important non-state actor within the international system of tobacco control, the FCA had the authority to hold governments and industry to account.

 The discursive power of civil society also helped the successful development of the FCTC. The FCA and other NGOs consistently framed tobacco as an ‘emergency’ public health issue requiring firmer action, and that the protection and promotion of public health must be the guiding principle for all decisions and actions of the negotiating parties. Tobacco companies attempted to divide developed and developing countries by arguing that tobacco control was an issue for only affluent countries, using the frame of a ‘First World agenda.’ The FCA worked to convince developing countries to treat tobacco control as a global issue, not just one for only affluent countries. The FCA used symbols to convey tobacco control as a global public health issue. The Death Clock, a large digital counter that displayed the number of worldwide deaths from tobacco-induced diseases since the beginning of the FCTC negotiation, was located at the plenary session entrance.

###  Fossil Fuels and the Divestment Movement 

Climate change is a major global health issue.^50,51^ The energy sector, particularly the fossil fuels industry, contributes the majority of the world’s greenhouse gas emissions.^52^ Transforming the energy sector from high to low carbon intensity is key to preventing further environmental degradation that will undermine social development and global health.

The global fossil fuel industry has incredible structural power, which has contributed to the failure of national and international governmental mechanisms to deliver on effective climate change mitigation measures.^53,54^ In the absence of government action, transnational non-governmental climate initiatives have emerged, looking beyond government for viable mitigation strategies.^55^

One such initiative is the global fossil fuel Divestment movement.^56^ Beginning in a US university in 2012, the university activist group – 350.org – launched its “go fossil free” campaign. The campaign spread across universities, and expanded into a global networked movement of 350.org, its affiliates and numerous other groups, including local governments and faith-based organisations.

 Unlike other NGOs who use institutional mechanisms such as stakeholder consultations, participation in public–private partnerships and lobbying to influence government action, the Divestment movement focuses on discursive, moral and network power. The aim of the movement is to convince institutional investors to divest from companies that have substantial assets in fossil fuel extraction, and to reinvest in renewable energy alternatives. Ultimately, the goal is to create structural reform in the economy away from fossil fuels toward renewable energy sources.

 The campaign strategy does not focus on the development of a specific policy instrument nor is it based on an economic argument. It focuses deliberately on moral and political persuasion, and shifting institutional and social norms. By applying pressure for divestment, the intention is to generate a value shift not just among institutional investors but also the wider public. In creating this broad constituency of support for climate action, politicians, in theory, will be emboldened and initiate effective action by governments.

The Divestment movement has used multiple discursive strategies. The use of the frame ‘If it is wrong to wreck the climate, then surely it is wrong to profit from that wreckage’ is designed to appeal to the ethics or morals of investors. Key to this moral strategy is the focus on institutions with social roles, such as pension funds and university endowments. They are shamed if they do not invest ethically. The movement has attempted to influence attitudes to fossil fuels by framing producer companies as pariahs, calling them ‘immoral, unscrupulous, intransigent, and as unnecessary to a future low-carbon economy.’^58^

 Importantly, the Divestment movement does not operate in a vacuum. There has been a rapid increase in climate change awareness at an institutional level. The endorsement of divestment as a strategy for climate change mitigation by inﬂuential individuals and organisations, such as the World Bank President, the International Monetary Fund, the United Nations Framework Convention on Climate Change, Governor of the Bank of England and Chairman of the G20’s Financial Stability Board and the Deutsche Bank, provides legitimacy to the movement. This legitimacy among influential stakeholders converts the power of the networked movement into authority, and this authority is key to influencing action and therefore amplifying the movement’s impact.

While there is a long way to go in transforming the energy sector, the reach and impact of the Divestment movement is large and rapid. From 181 institutions and $50 billion worth of assets committed to divestment at the end of 2013, to more than 1000 institutions with over $7.9 trillion in assets committed to divest from fossil fuels in 2018.^57^ There are pledges to divest in 37 countries and include major capital cities, mainstream banks and insurance companies, massive pension funds, faith groups, cultural, health, and educational institutions.

## Lessons for Healthy and Sustainable Food Systems

 In the previous section, the three cases describe different forms of power expressed across a diversity of governance approaches and sectors. The three cases reveal different combinations of strategies used by public-interest health and non-health sector actors to advance their interests in the face of incredible corporate power. This section explores lessons from the cases that will help public-interest actors enable the necessary structural transformations in the global industrial food system towards healthy and sustainable food systems.

###  Shared Interests and Goals

 While the overarching aim in each of the cases described previously was to recalibrate the power and influence of corporate actors (pharmaceuticals, tobacco and fossil fuel industries), the public-interest actors had a specific goal that they were working towards. In the case of access to medicines, the goal was a trade agreement that included legally binding text that ensured governments could take measures to protect public health, including the domestic manufacture of cheaper generic medicines. With tobacco, it was the establishment of a global regulatory treaty on tobacco control, including specific rules about engagement with the tobacco industry (Article 5.3), and with the divestment movement the goal was to stigmatize the fossil fuel industry and get institutional investors to divest from fossil fuel companies.

A key challenge for the healthy and sustainable food systems movement is the lack of a *specific shared goal*. The global food systems actor landscape is complex.^62^ In addition to food corporations, it involves national governments and different government departments; civil society groups; NGOs; the United Nations (UN) system; multilateral development banks and financial organizations; philanthropic organizations; and research institutes. Some of these actors are interested in economic goals, some focus on undernutrition, others on excess consumption, obesity and diet-related NCDs,^63^ and some focus on sustainable production.

Encouragingly, articulation of a shared goal has started among nutrition NGOs and some governments in relation to restrictions on marketing of food to children; nutrition labelling, and fiscal and pricing policy. For example, in Mexico an active civil society network worked together with a receptive government and policy advocate in the health minister to generate wide support for the successful introduction of a national level soda tax (a tax on sugar-sweetened beverages).^64^ The same networked strategy has been successful in passing soda taxes in seven local jurisdictions in the United States.^65,66^ While these soda tax successes may seem like a drop in the ocean, to the extent that they reduce consumption of harmful products they are a step in the right direction.

However, much more is needed if the rhetoric of ‘transform the global food system’ is to be operationalised. Strategically, it is important that public-interest food system actors reconcile their diverse interests and unite around specific goals. Building on the examples presented here, and the global food and nutrition evidence base, goals could be regulatory or normative, including (*i*) a framework convention on healthy and sustainable food systems, (*ii*) New trade treaties that ensure access to nutritious foods, and (*iii*) Divestment from harmful food industries.

###  Institutional Power

 The Doha Declaration and FCTC – the cases focused on binding international regulatory instruments – resulted from public-interest actors reclaiming structural power by taking a strong institutional approach, supported by persuasive discursive strategies. The existence of these international regulatory instruments returns structural power to states, enabling them to control outcomes based on their ability to shape the rules of the system and the relationship to corporations.

 In these cases, the institutional power of government and civil society actors was critical – they were able to make strategic use of intergovernmental mechanisms within the multilateral system to harness support for their issues, and they used formal institutional processes – committees, meeting agendas, stakeholder consultations, and lobbying – to push their interests. The civil society actors shopped their ideas around, moving from one institutional to another depending on which was most receptive to their agenda. In the case of access to medicines, civil society exploited intra-industry divisions to generate support for the Declaration from generic medicines companies.

There are important political discourses and policy windows at the multilateral level that are tantalisingly positive for sustainable food systems and global nutrition. Many of the seventeen UN Sustainable Development Goals address issues that would improve global nutrition and climate change.^59^ The political desire for action on global malnutrition is clear from the UN General Assembly resolution proclaiming the UN Decade of Action on Nutrition from 2016 to 2025.^60^ Food and nutrition actors must use their institutional power across the many mechanisms that exist in the UN and other international organisations to exploit this political opportunity.

 If the goal is some form of binding international treaty, as was the case with the Doha Declaration and the FCTC, this requires a strong lead by national governments, as the main constituencies of the treaties. Blocks of countries with healthy and sustainable food policies could table items for discussion in committees of the World Health Assembly, UN General Assembly, and other receptive venues to push the goal. Co-ordinated engagement and support from coalitions of nutrition, sustainable food systems and other supportive NGOs will help with lobbying and raising awareness about the issue and need for the particular type of action. International healthy and sustainable food system civil society actors must shop the ideas around, moving from one institution to another depending on which is most receptive to the agenda – there are plenty of venues to shift between eg, WHO, Food and Agriculture Organization, Scaling Up Nutrition, World Bank. As observed in the case of access to medicines, it will be important for civil society to find and exploit intra-food industry divisions to help generate support for the policy asks.

###  Ideational Power

While each of the three cases made use of ideational power, operationalising the Divestment case goal really centred on this form of power. The Divestment movement is not about the use of institutional processes and civil society engagement with government or the private sector. It is an example of pure non-state governance, with coalitions of NGOs operating as nodes within the ‘governance triangle’ formed by state, civil society and business actors.^67,68^ The Divestment movement is about shifting norms to catalyse the ‘energy revolution.’ It has established itself as a norm entrepreneur,^69^ using discursive power to ‘label a particular behaviour (carbon pollution) as morally reprehensible, and, by so doing, shift attitudes about climate change mitigation.’^56^ As we saw, the movement uses frames to shame morally reprehensible actors and persuade social-moral actors to change their practices. Institutional strategies, mainly local and international campaigning, direct action, and lobbying, are simply vehicles via which to deliver the discursive impacts.

There is a role for a similar movement within the healthy and sustainable food systems domain. The practices and products of some food industries are incredibly harmful for human and planetary health. We could use discursive power to label the behaviours of some TNCs as morally reprehensible, and help persuade social-moral actors to change their practices. For example, there is the opportunity to make use of the shifting sentiment within the investment community - the world’s largest fund managers are divesting from coal (https://www.afr.com/companies/financial-services/blackrock-dumps-thermal-coal-20200114-p53rd0). These same fund managers have major shares in food and beverage TNCs.

As observed in the Divestment movement and the FCTC, a lesson for a healthy and sustainable food systems movement is the importance of undermining corporations’ social license to operate through *holding them to account*. This can be done by shaming them through publicising their harmful business practices, or by using awards to applaud good behaviour and in doing so, extend or create inter- and intra-industry cleavages. There are already good accountability mechanisms in place for global nutrition including the International Network on Food and Obesity/NCD Research, Monitoring and Action Support. This research network has developed a monitoring system for food actions in relation to obesity and NCDs,^70^ including aspects of government food policy implementation and food industry actions.^71^ As recommended in the Lancet Commission on Obesity,^11^ this could be expanded to create a strong accountability system for healthy and sustainable food systems.

###  A Global Coalition

 In each of the three cases, the ‘structurally weak’ actors organised around a concerted campaign (access to medicines, tobacco free, go fossil free) focused on the shared goal. In addition, transnational advocacy networks emerged as important vehicles for change. It became clear that domestic NGOs and civil society groups could not resolve the problems through appealing just to national governments. In each case, political and policy entrepreneurs instigated global networking, believing that networks would help advance their interests. These networks worked to influence agendas, change the discourse, change institutional procedures, and ultimately change state behaviours and policy. Perhaps essential to the success of a healthy and sustainable food systems movement is the creation of transnational coalition of actors, identifying potential partners and new foot soldiers in the quest for a nutrition and environmental-oriented approach to food (eg, linking nutrition to climate issues to broaden coalitional base), organised around a clear campaign rallying cry.

## Conclusion

 Examining power dynamics reveals the structural, institutional and ideational factors that constrain or enable different actor’s behaviours, and the ways in which different actors navigate and change these factors to pursue their goals. Each of us concerned about good nutrition, human health and environmental sustainability are model mongers – using our agency to mobilise and work in different ways and in different venues towards a vision of healthy and sustainable food systems. We have seen the good that regulatory instruments including trade and investment agreements and global health treaties can do when reimagined towards the public interest. This highlights the importance of utilizing the variety of institutional processes that exist to government actors, and their supportive non-government counterparts, who on a daily basis use regulatory tools that make, generally, incremental change to systems that are each essential for nutrition and the environment. Often however regulatory and policy instruments are designed in ways that give more power to the already powerful. Processes of ‘political empowerment’ become important, whereby people, organisations, and nations gain control over the decisions that affect them and their shared vision. The social movements within each of the cases showed the importance of NGOs and civil society groups, working with like-minded organisations to mobilise and advocate for direct and indirect action that will rebalance the power of the corporations. Sometimes, this can be quite radical. When people feel betrayed by institutions they may turn against the political system. But people do not give up hope. The rise of their political consciousness finds expression outside of traditional arrangements in subcultures and countercultures. The Divestment movement is a powerful example of this.

The point of this paper is to highlight that things can and do change and that there is no one perfect way. Rebalancing the existing power inequities within the corporate food regime and achieving positive nutrition and environmental outcomes depends on networked combinations of different approaches rather than grabbing at only one lever of influence.^72^ Despite long odds, recalibrating the power inequities in the global corporate food regime may be possible through articulating an ambitious shared vision; coalition building; strategic use of institutional processes including forum shopping; social mobilization among like-minded and unusual bedfellows, and organized campaigns; the strategic use of political and policy entrepreneurs, and compelling issue framing.

## Ethical issues

 Not applicable.

## Competing interests

 Author declare that she has no competing interests.

## Author’s contribution

 SF is the single author of the paper.
